# In Search for Factors that Drive Hantavirus Epidemics

**DOI:** 10.3389/fphys.2012.00237

**Published:** 2012-07-10

**Authors:** Paul Heyman, Bryan R. Thoma, Jean-Lou Marié, Christel Cochez, Sandra Simone Essbauer

**Affiliations:** ^1^Epidemiology and Biostatistics, Research Laboratory for Vector-Borne Diseases, Queen Astrid Military HospitalBrussels, Belgium; ^2^Diagnostic Laboratory Division, Bundeswehr Institute of MicrobiologyMunich, Germany; ^3^Regional French Forces Medical CommandToulon, France; ^4^Department Virology and Rickettsiology, Bundeswehr Institute of MicrobiologyMunich, Germany

**Keywords:** Belgium, France, Germany, hantavirus, HFRS, NE, biotic factors, abiotic factors

## Abstract

In Europe, hantaviruses (*Bunyaviridae*) are small mammal-associated zoonotic and emerging pathogens that can cause hemorrhagic fever with renal syndrome (HFRS). Puumala virus, the main etiological agent carried by the bank vole *Myodes glareolus* is responsible for a mild form of HFRS while Dobrava virus induces less frequent but more severe cases of HFRS. Since 2000 in Europe, more than 3000 cases of HFRS have been recorded, in average, each year, which is nearly double compared to the previous decade. In addition to this upside long-term trend, significant oscillations occur. Epidemic years appear, usually every 2–4 years, with an increased incidence, generally in localized hot spots. Moreover, the virus has been identified in new areas in the recent years. A great number of surveys have been carried out in order to assess the prevalence of the infection in the reservoir host and to identify links with different biotic and abiotic factors. The factors that drive the infections are related to the density and diversity of bank vole populations, prevalence of infection in the reservoir host, viral excretion in the environment, survival of the virus outside its host, and human behavior, which affect the main transmission virus route through inhalation of infected rodent excreta. At the scale of a rodent population, the prevalence of the infection increases with the age of the individuals but also other parameters, such as sex and genetic variability, interfere. The contamination of the environment may be correlated to the number of newly infected rodents, which heavily excrete the virus. The interactions between these different parameters add to the complexity of the situation and explain the absence of reliable tools to predict epidemics. In this review, the factors that drive the epidemics of hantaviruses in Middle Europe are discussed through a panorama of the epidemiological situation in Belgium, France, and Germany.

## Introduction

Hantaviruses (*Bunyaviridae*) are carried by rodents, insectivores, and – as recently confirmed – by bats (Kim et al., 1994; Weiss et al., 2012) and transmitted to humans by inhalation of infected excreta (Heyman et al., 2009c). So far, only some rodent-borne hantaviruses have been found to be pathogenic to humans. The relationship between the rodent population density, the hantavirus prevalence in the rodent population, and the number of human hantavirus cases, including the hemorrhagic fever with renal syndrome (HFRS) cases in Eurasia, has been suggested worldwide (Tersago et al., 2011a). It is thus important to know whether or not hantavirus-carrier rodent populations are peaking or not. The factors that drive rodent population dynamics are of prime importance for predicting hantavirus epidemics (Linard et al., 2007a,b; Jonsson et al., 2010), but at the same time highly complex and largely unknown (Krebs, 1999).

The annually recorded numbers of clinically apparent hantavirus infections in Europe has been steadily increasing during the last 20 years (Heyman et al., 2011). In general, the awareness of public health authorities and the availability of diagnostics is supposed to have improved (Faber et al., 2010; Heyman et al., 2011), but one of the most important reasons might be that we have been ignoring a historical truth: human well-being and good health depends in the long-run on the stability of earth’s ecological and physical systems. It was easy to overlook or ignore this dependency in the nineteenth and twentieth century, when the human species began to grow exponentially, its environment was increasingly becoming urbanized and industrialized and natural systems became increasingly under pressure (Ramalho and Hobbs, 2012).

The human impact on ecosystems is indeed immense; man-made agricultural ecosystems dominate much of Europe’s landscape and, due to the intensification of agriculture and subsequent use of fertilizers and pesticides, biodiversity in particular has changed significantly in almost all agricultural areas (Sanchez et al., 2011; Shochat and Ovadia, 2011). The vast majority of farmland wildlife has suffered greatly and bird populations have decreased by 50% or more since the 1980s in Europe (Donald et al., 2006). Recently, the reduced use of both pesticides and fertilizers and environmental friendly farming (e.g., organic farming) mark positive changes that can be seen across Europe (Noyes et al., 2009). Forest ecosystems in Europe have also experienced dramatic declines, deforestation has however been reversed or management has changed in the last two decades and in some areas forests have been expanded significantly (Bezirtzoglou et al., 2011). Moreover, the often overlooked mountain ecosystems possess a high diversity of habitats and species and are important for water supply and its regulation toward lower altitude areas, but are also especially vulnerable to impacts from changes in agriculture, infrastructure, tourism, and climate (DeGraaf et al., 1992; Huitu et al., 2003).

Urbanization was and still is a significant factor that is changing Europe’s biodiversity, mainly because of the inevitable rural to urban land-use change (Franklin, 1993). Urban ecosystems are therefore almost never integrated into wider biodiversity considerations, in the past two decades the increasingly renewed contact of people with nature (de-urbanization) has been found to relieve urban stress and to help fight climate change through increased awareness, but it has also made people – through increased risk for contact with wildlife – more vulnerable for zoonotic pathogens (Franklin, 1993). Adverse health effects need however not be exclusively related to infectious diseases. One of the most striking recent examples of detrimental health effects related to climate was the heat wave during the summer of the year 2003, which was probably the hottest summer in Europe for 500 years. Between 20,000 and 40,000 heat-related deaths occurred across Europe in August 2003 alone (Schär et al., 2004; Laaidi et al., 2011).

Ironically, while human pressure onto the environment had detrimental effects for the entire ecosystem, recent observed change for the better might – in collaboration with changes in human behavior and climate – increase the chances for infectious diseases transmission. Their reservoirs and vectors – ticks, fleas, insects, and mammals mostly – are in general not amongst the many species that appear on the IUCN red list^1^. But although rodents – the reservoirs for hantaviruses – are generally not regarded as threatened mammals, there is ample historical evidence of the extinction of several rodent phylogenetic lineages. Rodent species represent about 50% of mammalian extinctions in the last 500 years (Amori and Gippoliti, 2003). Rodents will probably remain unpopular, despite increasing evidence that many rodent species in fact sustain ecosystems structures and functions (Amori and Gippoliti, 2003).

As this paper aims to define factors that drive hantavirus epidemics, an important question is to what extent certain parameters influence hantavirus activity and whether these parameters occur solely in one region or on multi-country level. Perhaps the most difficult question is how factors that facilitate and those that inhibit the hantavirus transmission mechanism interact and what the net outcome of this equation is and will be in the future. The following chapters summarize the present knowledge on hantaviruses and disease transmission in Belgium, France, and Germany. These three countries were chosen as examples for discussing the epidemiology, and possible factors having an impact on changes in the oscillations of hantaviruses in Middle Europe.

## The Hantavirus Situation in Belgium

Belgium is situated in the temperate deciduous forest biome, it consists of three main geographical regions; a highly industrialized coastal plain in the northwest and central plateau, and the mainly forested Ardennes in the southeast. Belgium’s highest point (694 m) is located in this region. The Ardennes extend into N-France and in W-Germany.

**Figure 1:**
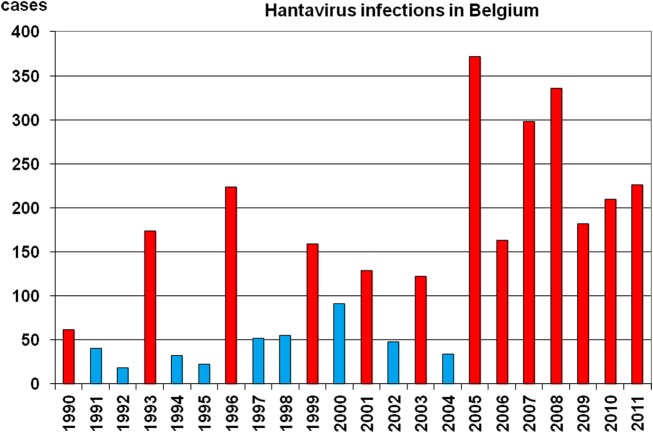
**Yearly number of hantavirus cases in Belgium as diagnosed by the Reference Laboratory for Vector-Borne disease, Brussels**. The epidemic years are depicted in red, the non-epidemic years in blue.

Although scattered data concerning clinically apparent human hantavirus infections are available from 1976 on in Belgium, this was only reliably and on a national level recorded from 1996 on by the Reference Laboratory for Vector-Borne Diseases (RLVBD) in Brussels, that was appointed National Reference Laboratory for Hantavirus Infections; the data were regularly reported to the Scientific Institute of Public Health (SIPH), the reporting organism to the public that is usually – but erroneously – cited as the source of the data (Figure 1). A total of 3,124 cases have been diagnosed according to the case-definition of the European Network for Imported Viral Diseases (ENIVD^2^) until end of 2011 in Belgium.

**Figure 2:**
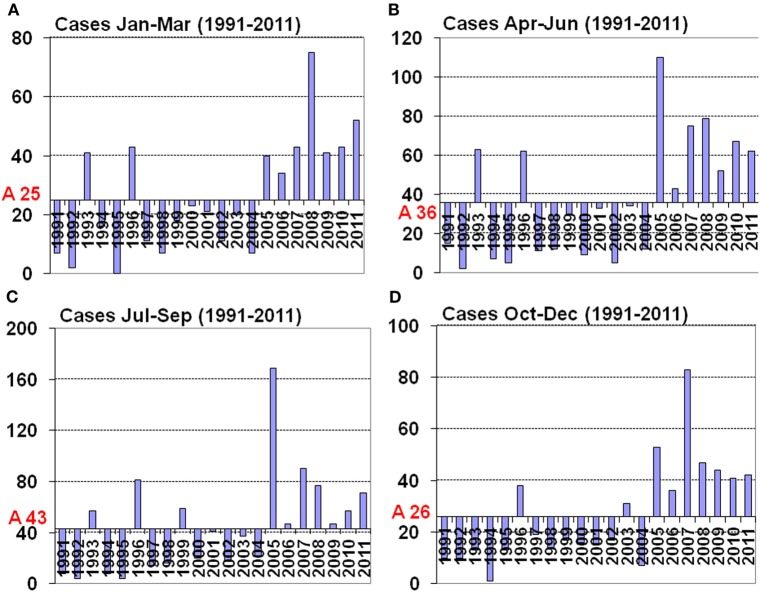
**Average of seasonal hantavirus cases and seasonal distribution (calculated as deviation from the average) in Belgium for the years 1991–2011**. **(A)** Months January to March with an average of 25 cases, **(B)** months April to June with an average of 36 cases, **(C)** months July to September with an average of 40 cases, **(D)** months October to December with an average of 26 cases.

Puumala virus (PUUV), carried by the bank vole (*Myodes glareolus*) is the only hantaviral serotype that is known to infect humans in Belgium, it induces a mild form of HFRS called nephropathia epidemica (NE); on average 150 cases occur yearly. From 2005 on there is a definite increase in the yearly number of NE cases in Belgium (Figures 2A–D). Although increased awareness and improved diagnostic tools were originally argued for the increasing number of yearly cases, the RLVBD data (Heyman, personal communication) show that the total number of serology demands is more or less constant between the years but that the number of submitted samples that showed positivity for hantaviral antibodies increased from 25 to 56%. Thus, increased awareness and improved diagnostic tools do not seem to be responsible for the increase in cases, a more targeted diagnostic strategy applied by clinicians is however possible. Under-diagnosis is probably also important as only 5–10% of the infected individuals display clinical symptoms (Heyman et al., 2009c).

From the data collected by the Belgian reference laboratory we learn that there existed –from 1993 on – a 3-year epidemic cycle up until 1999, between 1999 and 2005 a 2-year cycle occurred and from 2005 on all years – i.e., seven in total – showed an increased hantavirus activity (Figure 1). This unique hantavirus epidemiology pattern that only occurs in Belgium and not in the surrounding countries raises questions whether the parameters that drive hantavirus epidemiology apply only to Belgium or also to other European countries (Heyman et al., 2011).

Here we discuss a set of parameters that could drive hantavirus epidemics in Belgium.

### Carrier and host behavior

In the case of hantaviruses, rodents and insectivores act as carrier, humans and non-rodent mammals (cats, dogs, foxes, deer, boar, etc.) act as dead-end hosts. So far, only infected humans can become ill although no sufficient evidence exists to exclude other mammals as competent hosts (Zeier et al., 2005). In most cases the virus is transmitted from animal to human by inhalation of an aerosol of infected rodent excreta (Heyman et al., 2009c). In order to make this possible three main conditions must be met.

- The carrier must be present and be sufficiently numerous.
  There is sufficient evidence that the rodent population density in Belgium is directly related to the percentage of infected carriers in the population and to the number of infected hosts (Heyman et al., 2002b). Some hantaviruses can also spill-over to other habitat sharing rodent species (*M. glareolus* to *Apodemus sylvaticus* in the case of PUUV; Klingström et al., 2002; Heyman et al., 2009b). In some cases the secondary carrier species can be heavily infected and – although there is no proof provided – chances are that both primary and secondary carrier species could infect humans (Klingström et al., 2002; Heyman et al., 2009b; Schlegel et al., 2009).
- The host must have access to the habitat of the carrier or vice-versa.
  In order to infect the host, he must remain in the carrier habitat for a sufficiently long period of time and also be involved in activities that put the host in direct contact with the virus particles. Entering and cleaning long abandoned places (cabins, attics, cellars, etc.) or performing work that disturbs carrier nests (renovation, cleaning, etc.) or other activities that put the host in prolonged contact with the carrier’s habitat (camping, sleeping on the ground, military exercises, etc.; Linard et al., 2007a; Tersago et al., 2011a). This rule is also valid for non-human mammals although the involved species usually are predating on the carrier species (Dobly et al., 2012). The observation that about 85% of the hantavirus cases occur in the forested South of the country, where wood is commonly used for heating and building, may support this.
- The contact between aerosol and host must take place within a limited time frame, i.e., the virus must still be viable. In a minority of the cases direct contact with rodents through handling dead rodents or rodent’s nesting material of live rodents can play a role.

Although hantaviruses are RNA viruses that are considered to be vulnerable for various biotic and abiotic parameters once they are outside the carrier, studies have revealed that the virus can remain viable for an extended period of time (days to weeks) outside the carrier if conditions are favorable (Kallio et al., 2006a). This implies that it is by no means necessary to actually see or encounter rodents in order to become infected. Again, the more common manipulation and use of wood in south Belgium may support this (Campioli et al., 2009). The observed change of the climatic conditions in Belgium with warmer winters and wetter summers (Tricot et al., 2009) might favor the survival of the virus in the environment and contribute to the higher number of cases.

### Rodent ecology

During the glacial events of the Quaternary in Europe, deciduous forests were generally confined to the Mediterranean peninsulas (Deffontaine et al., 2005). Temperate forest mammal species such as bank voles shifted their range according to their habitat, thus surviving the glacial maxima in the Mediterranean peninsulas (Taberlet et al., 1998; Deffontaine et al., 2005). Interglacial and postglacial recolonizations of central and northern Europe, by plants and tree accompanied by – amongst others – rodents, therefore originated from these refuges (Taberlet et al., 1998; Michaux et al., 2005).

Depending on food availability, predation and environmental characteristics, and stress, various endemic rodent species display more or less regular cyclic population changes. Whether population numbers and cycles of endemic rodents in Belgium have changed is not known but with the cutting of forests and the human population increase since Medieval times it seems reasonable to assume that nowadays rodent populations are a fraction of what once was. Rodents often were a serious economic and public health problem; one of the most striking examples were the consecutive plague events in Europe between the fourteenth century and the last occurrence in Marseille in 1720 (Duchene and Contrucci, 1998) that killed in certain regions up to 30% of the population. A curious side effect of the significant human population decrease was that from about 1,350 on CO_2_ levels in the atmosphere dropped due to reforestation as less wood was needed for building and heating for the decimated population (Ruddiman, 2003).

**Figure 3:**
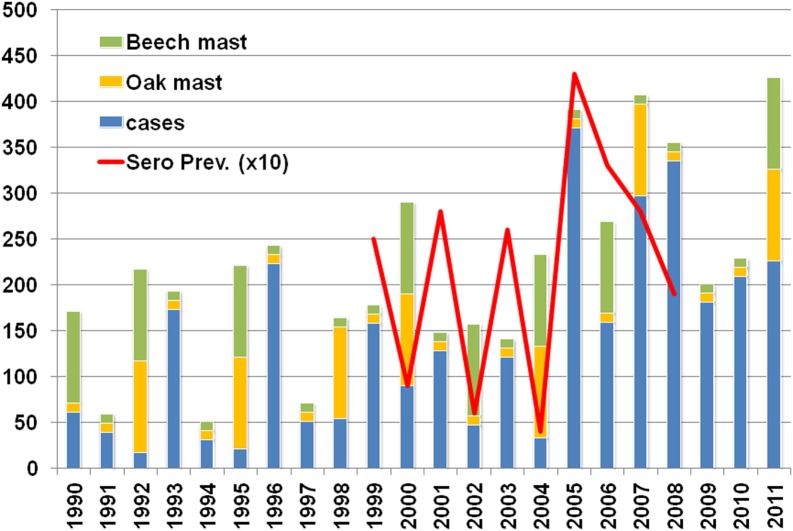
**Oak (*Quercus robur*) and Beech (*Fagus sylvatica*) mast years, hantavirus cases, and seroprevalence in *M. glareolus* during the period 1999–2008**.

Bank vole populations in Belgium indeed show cyclic population density changes (Heyman and Saegerman, 2009). A 10-year (1999–2008) rodent surveillance project organized by the Belgian Ministry of Defense with rodent trapping on 15 different sites demonstrated that between 1999 and 2005 fluctuations in trapping success (and thus rodent population density) correlated with hantavirus prevalence (measured by detection of IgG antibodies) and with the relative number of human cases. From 2006 on, however, this relation is much less clear, i.e., rodent population densities remain high in the absence of a mast event – in this case beech and/or oak – and virus prevalence remains higher than expected (Figure 3).

Although allegedly not critical for rodent cycles in the temperate deciduous forest zone, predation, or behavioral changes due to the presence of predators could also play a role in Belgium. Population densities of the various animals (mammalian and avian) that predate on *M. glareolus* are not well known, but – apart from population density increases of birds of prey species (Robinson and Sutherland, 2002), Population densities of *Mustelidae* (weasels) and foxes (*Vulpes vulpes*) are on the rise since the 1980s in accordance with bank vole population fluctuations (Vervaeke et al., 2003).

### Biodiversity

Due to intensive agriculture, pesticide, and insecticide use and urbanization, the mammal biodiversity in Belgium is in almost all habitats changed. Although 21 rodent and 6 insectivore species are endemic in Belgium (Wilson and Reeder, 2005), trapping data – as obtained in the forenamed 1999–2008 study of the Belgian Ministry of Defense – reveals that only five rodent (*Microtus arvalis*, *M. glareolus*, *A. sylvaticus*, *Apodemus flavicollis*, *Rattus norvegicus*) and two insectivore species (*Sorex araneus*, *Crocidura leucodon*) are readily captured in live traps. In the study Ugglan Special No2 traps (Grahnab, Gnosjö, Sweden) that are suitable for trapping the vast majority of *Apodemus*, *Myodes*, *Microtus*, *Sorex*, and *Crocidura* species were used. *Talpa europaea* is also common but requires a specific trapping strategy due to its subterranean life. In Belgium, *M. arvalis* is the carrier for Tula virus (TULV; Heyman et al., 2002a), *M. glareolus* carries PUUV, and *A. sylvaticus* is a spill-over host for PUUV (Heyman et al., 2009b), *A. flavicollis* carries Dobrava virus (DOBV) but this virus is so far not found in Belgium, *R. norvegicus* carries Seoul virus (SEOV) which is readily present in Belgian rural brown rats (Heyman et al., 2009a) but to this point no human cases have been detected. The remaining 22 species are rare (Pucek, 1989). It is generally regarded that high biodiversity has a diluting effect for pathogen transfer to a single host (Mills, 2006; Peixoto and Abramson, 2006; Keesing et al., 2010), deteriorating landscape and subsequent decrease of biodiversity could thus favor hantavirus infections. The disturbance of trophic interactions is considerable for virtually all species (Olsson et al., 2010).

### Landscape

Endemic species are amongst the most affected if an original forest is converted into an anthropogenic habitat. The native vegetation is in most cases partially replaced by non-native or even invasive plant species by gardening activities. A matrix of altered habitats enclosing forest remnants in human dominated landscapes may be inhospitable for wildlife in general. Endemic species are in this case under increasing pressure from non-native or invasive species and both invasive species and generalist species tend to survive the changes (Umetsu and Pardini, 2007).

Habitat fragmentation is also regarded as a possible triggering factor for biodiversity loss (Fahrig, 2003). The landscape fragmentation and absence of corridors can also be responsible for creating so-called islands where rodent populations display the typical Island syndrome features (differences in demography, reproduction, behavior, and morphology when compared to normal populations; Adler and Levins, 1994; Eccard et al., 2011). Landscape changes may thus partially explain the success of the bank vole in virtually all habitats in Belgium and the increasing risk for hantavirus infections. Unfortunately, the intensive agriculture techniques applied in Belgium turned the landscape into a green desert, where wildlife or wild plant species have no place (Peeters et al., 2006, 2007). A significant disturbance of trophic interactions can occur and only generalist and invasive species may profit (Erhard, 2009; Jonsson et al., 2010).

### Climate

Climate change is often referred to as one of the main causes of the increase in magnitude and amplitude of the hantavirus infection curve in Belgium, the problem however is that climatic events are often locally, vaguely, and qualitatively defined (Krebs and Berteaux, 2006). The average temperature in Belgium is defined by two parameters: the distance from the sea and the altitude of a certain location.

The fourth IPCC report (The Core Writing Team, 2007) stipulates that “warming of the climate system is unequivocal, as is now evident from observations of increases in global average air and ocean temperatures, widespread melting of snow and ice and rising global average sea level,” albeit that changes in temperature and rainfall are predicted to be more pronounced at higher latitudes and along the equator than in Western Europe.

**Figure 4:**
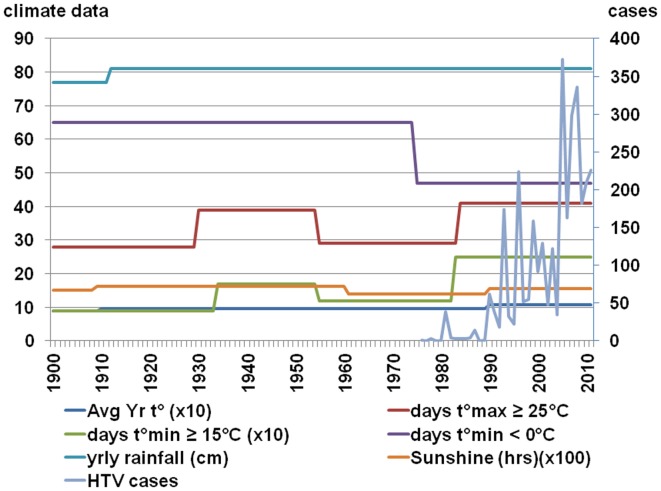
**Climatic conditions in Belgium according to Tricot et al. (2009)**.

The Belgian Royal Meteorological Institute is monitoring the climatic parameters in Belgium since 1833 and is currently relying on a network of more than 250 daily reporting stations throughout the country. In its 2009 climate report, detailed information is made available concerning the tendencies of the Belgian climate in the past century (Tricot et al., 2009). The average temperature has risen with about 2°C since 1833, but this happened in two rather abrupt stages (Figure 4); the first significant increase took place around 1910 with primarily a rise in maximum temperature, the second around 1990 with a rise of both maximum and minimum temperatures. Both times the increase was about 1°C. The first frost day happened around the 30th of October from 1900 up until 1955, than an abrupt delay of – on average – 10 days occurred. The last frost day shifted from around the 22nd of April to 06th of April with – again – two abrupt changes in 1930 and 1980 (Figure 4). The number of days with at least 25°C and the number of days with temperatures of at least 15°C showed two warmer periods, i.e., from 1930 to 1950 and from 1983 until present. Although the number of days with >25°C has increased, there is no evidence that the number of sunshine hours (related to UV index) has increased as this is associated with cloud cover which is difficult to measure. The UV index increase – important for inactivation of RNA viruses – is thus not established. Finally, the yearly rainfall of on average 770 mm increased around 1910 to 810 mm. The Belgian climate has changed during the twentieth century. Two sudden temperature increases – one in the first half of the century, the other during the 1980s – were noted, the heat wave frequency has increased in the 1990s, the frequency of cold waves decreased in the 1970s.

It should also be taken into account that – even in a country like Belgium where the greatest distance between its borders is no more than 300 km – climatic conditions sometimes significantly vary between the coastal region and the inland regions. There exists an average temperature difference of 2.5°C between the coast region and the Ardennes region, a temperature decrease of approximately 0.6°C per 100 m of altitude increase is observed^3^.

Thus, explaining the increase in the number of NE cases in the last decade (2001–2011) in Belgium just by climate change is difficult because the key years for changes in hantavirus activity were 1999 and 2005; if milder climatic conditions caused the increase in cases, these events should have been visible much earlier as the key years for climatic changes in Belgium occurred long before (Figure 4). If – even in the absence of the necessary diagnostic tools – hantavirus epidemics had taken place before the first reliably recorded outbreak of 1995–1996 (Heyman et al., 1999) they would not have gone unnoticed given the sometimes severe clinical course of hantavirus disease, i.e., acute renal failure (ARF) and/or acute respiratory distress syndrome (ARDS). In Russia for instance, hantavirus infections were already diagnosed based on clinical features as early as 1935 (Sirotin and Keiser, 2001).

### Mast

“Mast” is a term used to describe the seeds of shrubs and trees that are eaten by wildlife. “Hard mast” refers to nuts of beech and oak, whereas “soft mast” refers to leaves, flowers, and berries of a variety of species. A number of different levels of mast are used such as full, half, dispersed, or deficient but these criteria are not always quantitatively measured. The mast theory – although recently claimed by some in Belgium – is by no means new. Charles Elton (1900–1991) was the first to systematically study animals in their natural habitats and their interactions with their surroundings (Elton, 1933).

Hundreds of animal species use mast as staple food. No less than 171 species (16 amphibian, 9 reptile, 102 bird, and 44 mammal species) are known to depend on it in beech or oak stand habitats (DeGraaf et al., 1992). The interspecies competition already suggests the use of alternative food sources for mammals as they can also feed on soft fruits, plant, or insect species (Bozinovic, 1997; Elkinton et al., 1998).

**Figure 5:**
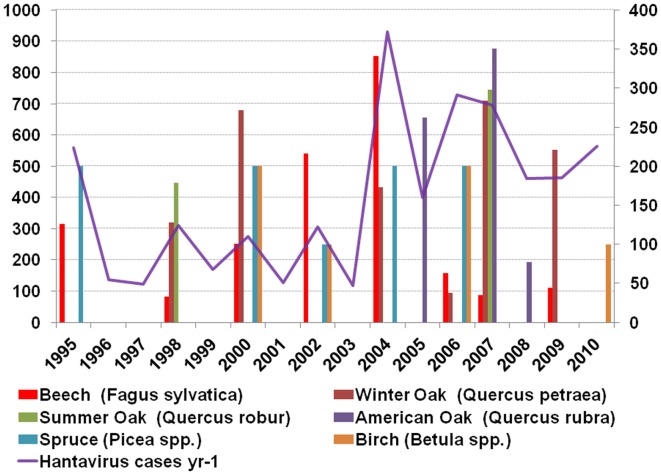
**Masting events and masting species (Source: Comptoir forestier de Wallonie)**.

The connection between mast and hantavirus infections was – in Belgium – already suggested in 2001 (Heyman et al., 2001) and re-examined recently (Tersago et al., 2009; Figure 5). The scarcity of detailed mast data, regional mast data, the short time frame for which detailed data are available and the complete lack of data on other than oak and beech species that have a cyclic or yearly masting event limits the value of the observations. Only oak and beech mast were involved in the various studies but closer examination shows that – in the habitat of *M. glareolus* – between 11 and 33 (November to February) and 95–1,292 (March to October) flowering plant species^4^ are available as food source (Kollmann et al., 1998). As beechnuts and acorns are only available between October and January and are also the staple food for large mammals like *Sus scrofa* (wild boar) or *Capreolus capreolus* (roe deer) and various bird species, it seems obvious that rodents need to rely on other food sources for most – if not the whole – of the year. Besides evidence for an animal-based food preference in spring (Eccard and Ylönen, 2006), it was also established that hard fruits only account for about 40% of the bank vole’s diet (Wereszcynska and Nowakowski, 2004). Bank voles are generalists and omnivorous, they eat insects, leaves, and soft fruits as well as hard fruits (Kollmann et al., 1998).

As far as oak and beech mast is concerned, Belgian data show that hantavirus epidemics are to some extent related, but only for the period between 1995 and 2007 (Tersago et al., 2011b). Not in favor of the role of beech and oak in hantavirus epidemics and probably the proverbial straw that breaks the camel’s back is the fact that both species only account for 9 and 17%, respectively, Norway spruce (*Picea abies*) represents more than 36% of the total tree stand composition (Campioli et al., 2009). Before 1995 mast did – to our best knowledge as reliable data are lacking – not correlate with hantavirus disease epidemics. After 2007, i.e., the last 3 years (2008, 2009, 2010) were no mast years but hantavirus activity was nevertheless high (Figure 5). In fact, only the 1993, 1996, and 2005 epidemics correlate with a previous oak-beech mast event, 2001 should have been an epidemic year, after the oak-beech mast event of the year 2000, but was not. And finally, all years after 2005 – six in total – were epidemic years in the absence of oak-beech masting events (Figure 5). From Figure 5 it can be concluded that any mast event can provide sufficient food for rodent populations to peak and induce a hantavirus epidemic in the next year. Thus, not only beech or oak trigger these events. Another observation that does not support the Belgian “mast theory” (Clement et al., 2011) is the fact that mast is considered to take place on a large scale, even on sub-continental level (Kelly et al., 2008; Olsson et al., 2010), the variation of epidemic years in neighboring countries however does not support this (Heyman et al., 2011). Even locally, i.e., in Belgium, masting is not present at the same time in all regions; the 2011 mast of oak-beech is present in the Flanders region, the North and South – but not in the middle part – of the Wallonia region, this does not mean however that other plant species did not display mast. One other intriguing fact is that although hantaviruses are supposed to co-evolve with their carriers for millions of years – clusters of hantavirus disease (NE, ARF) were not reported before the 1980s (van Ypersele de Strihou et al., 1983). Even in the absence of diagnostic tools the syndrome caused by hantavirus infection should have been noticed and periodically, i.e., the year after a mast event, reported by clinicians.

## Epidemiological Features in France

### Hantavirus species in France

In France, PUUV is the most prevalent hantavirus species (Artois et al., 2007). As already explained in the Belgium chapter, this virus is closely associated with its natural reservoir host, the bank vole *M. glareolus*, but the transmission to other dead-end hosts (including humans) remains possible (Deter et al., 2008a). During a survey in France, PUUV seropositive wood mice (*A. sylvaticus*) were detected during the peak of prevalence in bank voles (Sauvage et al., 2002). PUUV was also serologically found in 0.3% (9/263) of the montane water voles (*Arvicola scherman*) captured in the Jura region during 2002, 2003, and 2004 (Charbonnel et al., 2008). Genetic analysis of S and M segments of French PUUV strains revealed their proximity with strains circulating in Belgium and Germany and also in Slovakia (Plyusnina et al., 2007).

Seoul virus, another pathogenic hantavirus, was detected in 2003 in rats captured near Lyon (Heyman et al., 2004). Using reverse transcriptase polymerase chain reaction (RT-PCR) and sequencing, two wild *R. norvegicus* were found infected with a SEOV strain, most closely related to an Indonesian and a Cambodian wild-type strain (Heyman et al., 2004). Human sera positive for SEOV antibodies have been reported in Europe using highly cross-reactive immunofluorescence assays, but have never been confirmed by reliable methods such as neutralization. Nevertheless, as opposed to Belgium, SEOV-neutralizing antibodies have been detected in the convalescent serum of a French patient suffering from HFRS (Lundkvist, personal communication).

More recently, TULV has been identified using RT-PCR in one vole (*M. arvalis*) trapped in the Department of Jura, close to the Swiss border (Artois et al., 2007). This report was confirmed later, with 6.5% (11/170) of *M. arvalis* found seropositive for TULV (Deter et al., 2008b). Based on partial sequence of the M segment, TULV strains circulating in France were found to form a distinct lineage, close to strains isolated in central and Eastern Europe (Plyusnina et al., 2007). TULV has not definitely been linked to human disease (Heyman et al., 2011).

To date, DOBV, responsible for severe cases of HFRS, has not been reported in France, although one of its carriers, *A. flavicollis* is present (Mitchell-Jones et al., 1999).

### Geographical distribution

**Figure 6:**
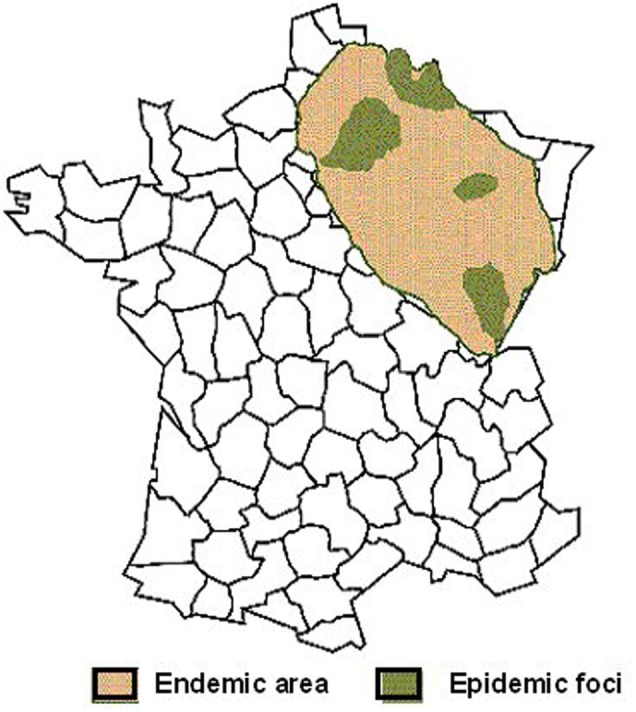
**Geographic distribution of human Puumala hantavirus infections in France (Source: French Ministry of health)**.

In France, most of the HFRS cases are reported in the northeastern quarter of the country (Augot et al., 2006; Figure 6). In this endemic area, foci of hot spots are well known, more particularly in the Ardennes department, near the Belgian border, which historically accounts for 30–40% of the total number of human cases in France (Sauvage et al., 2002).

**Figure 7:**
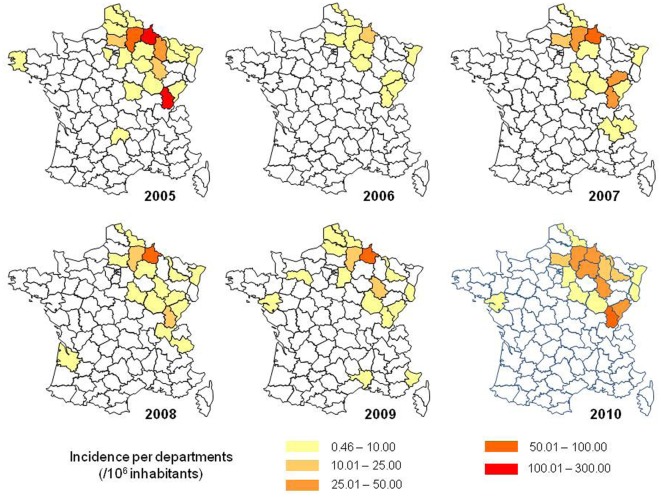
**Incidence rate of hantavirus infections in France, according to the department of residence of the patients (Source: Institut de Veille sanitaire, IVS)**.

As illustrated in Figure 7, endemic foci stay rather stable over years, without a real trend for extension. In addition to the historical department of the Ardennes, a new hot spot appeared in the department of Jura, bordering Switzerland, since the epidemic of 2005 (Figure 7).

In fact, *M. glareolus* is distributed in the whole country, excepted along the Mediterranean coast (Mitchell-Jones et al., 1999), in a much larger area than the endemic area for HFRS.

### Infection in rodents

Several studies have been carried out, in the endemic area, in order to assess the prevalence of PUUV in *M. glareolus*, and to elucidate the dynamics of circulation of the virus in its wild reservoir and thus estimate the risk for human beings. To date, no results have been published for surveys outside the endemic area.

As shown in Table 1, the seroprevalence levels are highly variable, between areas and in the same area, according to the year and the season (Augot et al., 2008).

**Table 1:** **Seroprevalence study of Puumala virus infection in bank vole (*Myodes glareolus*) populations in France (95% confidence interval in brackets)**.

Location	Period	Seroprevalence (PUUV positive/investigated rodents)	Confidence interval (%)	Reference
Jura	2004 and 2005	9.0% (12/133)	4.2–13.9	Deter et al. (2008a,b)
Ardennes	2000–2002	22.4% (204/912)	19.7–25.1	Augot et al. (2008)
Ardennes	2004 and 2005	37.6% (291/773)	34.2–41.1	Augot et al. (2006)
Ardennes	2008 (autumn)	13.5% (37/274)	9.5–17.6	Guivier et al. (2011)
Ardennes	2008 (September–October)	11.8% (37/313)	8.2–15.4	Salvador et al. (2011)

Transmission among the rodent populations occurs via excreta, i.e., saliva, urine, and feces, directly or indirectly, probably through aerosols. The voles may become infected through the sniffing of contaminated excreta marks, even if they avoid direct encounters. The virus remains active outside the host, allowing indirect transmission, without physical contacts with infectious rodents. PUUV can survive and remain infectious for 15 days at room temperature, and probably much longer in cold and moist environments (Kallio et al., 2006a). Vertical infection seems improbable (Deter et al., 2008b) as well as the contamination from mother to progeny. Indeed, juveniles are protected during 2 or 3 months because of the transfer of maternal antibodies (Bernshtein et al., 1999) and the dispersal from the natal site starts before. However, in case of high densities, the dispersal of bank voles is delayed until next spring and juveniles can get infected from their contaminated mother, after the disappearance of the maternal antibodies (reviewed in Deter et al., 2008a).

Usually, the adult males are more often infected than females and juveniles or sub-adults for behavioral and/or physiological reasons (Bernshtein et al., 1999). In the Ardennes, the antibody prevalence rate also increased with age (weight) of the bank voles, suggesting that horizontal infection could be important (Augot et al., 2006). Later, the same author concluded that a strong positive correlation is usually found between seroprevalence and age (estimated with the weight) of the rodents (Augot et al., 2008).

The effect of sex on the prevalence of infection remains unclear, as in some French studies males are more often infected (Deter et al., 2008a) but in others, no significant difference is found (Augot et al., 2008). Nevertheless, the behavior between males and females differs. Female territories are smaller than those of males, especially during the reproductive season (Mazurkiewicz, 1994), and males are much more tolerant for territorial overlap (Augot et al., 2008). Compared to females, when males leave the nest, they traverse greater distances and could introduce the virus in virus-free rodent populations (Augot et al., 2006). Behaviors and social features are critical for understanding the persistence and the spread of hantavirus among rodents populations.

The fluctuations of PUUV prevalence in the bank vole populations have generated multiple hypotheses. Some authors consider prevalence is linked, sometimes with a delay, to the density of the rodent reservoir (Davis et al., 2005). Other authors found that the seroprevalence is not always linked to the density of rodents but usually, higher numbers of seropositive bank voles are captured when the populations decrease from a peak year (Kuenzi et al., 1999; Augot et al., 2008). In other surveys, PUUV seroprevalence and abundance of rodents are weakly linked (Augot et al., 2008).

In France, as more generally in Atlantic and continental Western Europe, mast events seem to be linked to hantavirus epidemics (Heyman et al., 2011). Here in comparison with Belgium several studies have shown that masting in the previous autumn increases food resources and improve the winter survival of rodents which start to breed earlier and can have a second litter, bringing high densities of rodents early in summer (Sauvage et al., 2002; Vapalahti et al., 2003). It appears that animals from the first litter, born in late winter or early spring develop rapidly and become fertile the same year whereas animals from the second litter, born late spring or mid-summer, become fertile only next year. The seroprevalence was significantly higher in animals from the first litter (48%) when compared to the second litter (8%; Augot et al., 2006). The high proportion of susceptible animals could favor a high level of PUUV circulation and a massive shedding of virus in the environment, source of rodent, and human infections (Bernshtein et al., 1999; Escutenaire et al., 2002). In fact, the amount of virus shed during the first month of infection is far higher than during the consecutive chronic phase (Bernshtein et al., 1999; Sauvage et al., 2007). More than the global density, the number of newly infected voles, still in the acute phase of the infection, appears critical to lead to exceptionally high virus concentrations in the environment and thus human exposure and epidemics. In more stable populations, the low incidence of newly infected individuals could explain a lower contamination of the environment. This assumption could explain the presence of only sporadic cases outside the endemic area for hantavirus in France, whereas *M. glareolus* is distributed nearly in all the territory. In brief, epidemics could occur in areas where multi-annual fluctuations of bank vole populations induce at the same time a high number of infected rodents and high proportions of those rodents in the acute phase of the excretion.

As is the case for Belgium, hantavirus epidemics in France can probably not solely be related to mast cycles as outbreaks appear to be localized in foci when mast events are supposed to occur over large areas (Heyman et al., 2009c). The prevalence of the infection in rodents seems also linked to the habitat (Escutenaire et al., 2002; Olsson et al., 2005; Zeier et al., 2005). Forest environments seem more favorable for rodent infection than fragmented, heterogeneous landscapes as hedge networks (Guivier et al., 2011). Different factors can explain this difference. First, hedges are usually considered of a lower quality for food resources than adjacent forests (Guivier et al., 2011). Besides, hedge networks are more exposed to important variations in temperature, humidity, and UV radiations, altering the survival of the virus in the environment and the efficiency of indirect transmission (Guivier et al., 2011). In addition, the helminth community of bank voles is linked to the environment and was shown to be associated with PUUV infection. In particular, bank voles infested by *Heligmosomum mixtum* were more often PUUV positive (Salvador et al., 2011).

The risk for human infection seems to be strongly correlated with the prevalence of PUUV in populations of its reservoir host species (Salvador et al., 2011). But additional factors intervene as the survival of the virus in the environment may relate to temperature, humidity, and UV as well as human behavior, necessary to come into contact with the virus.

### Human infections

In France, the first clinical case of hantavirus infection was reported in 1977, in the forest of the Ardennes, near the Belgian border (Cousin, 1979). Nevertheless, the epidemics of “trench nephritis” described during world war one (Atenstaedt, 2006) were probably caused by hantavirus.

The national reference laboratory for hemorrhagic fevers (CNR) is in charge of hantavirus infection surveillance in France. The serological diagnosis relies on indirect immunofluorescence since 1982. In 1993, a confirmation with the research of specific IgM antibodies using an enzyme-linked immunosorbent assay (ELISA) has been added (Le Guenno et al., 1994). When a positive or a doubtful serological diagnosis is made by another laboratory, which is possible since 2002, a confirmation is systematically performed by the CNR, making the centralization of the results and the surveillance possible. In case of a positive laboratory result, a questionnaire is sent to the clinician of the patient in order to identify the risk factors. Later the Institut de Veille Sanitaire (IVS) analyses these data and provides public health recommendations.

**Figure 8:**
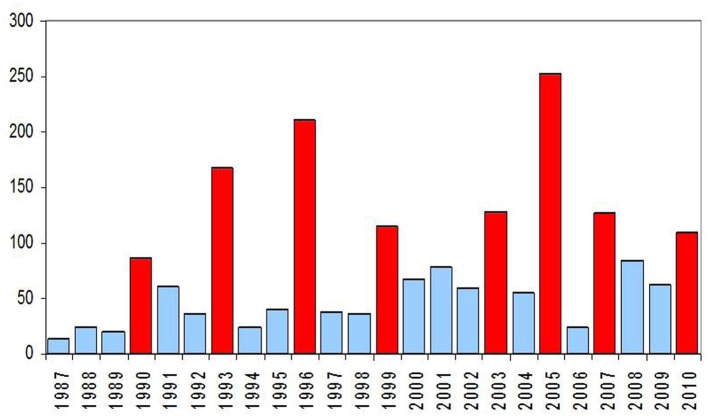
**Number of hantavirus infections in France during the period 1987 – July 2010 (data: Institut de Veille Sanitaire, IVS)**.

**Figure 9:**
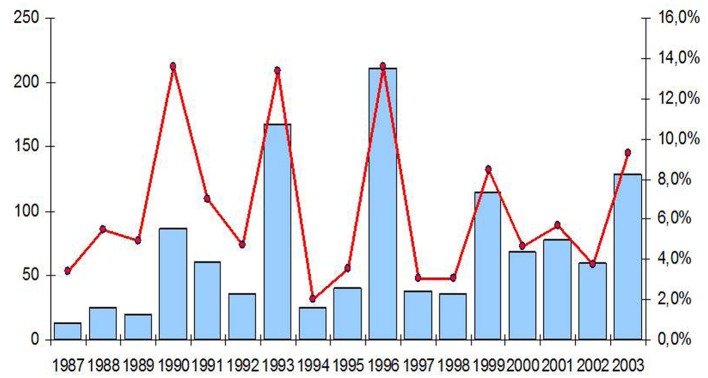
**Number (graph in blue) and proportion of positive results (curve in red) of hantavirus infections in France, period 1987–2003 (data: Institut de Veille Sanitaire, IVS)**.

The annual number of hantavirus infections reported in France from 1987 to July 2010 is shown in Figure 8. If 60 cases in average are diagnosed every year, with an increase during the last decade, the incidence varies greatly and epidemic years (in red in Figure 8) occur every 2–4 years. The increased number of cases reported in epidemic years is not a consequence of a better physician awareness leading to a greater number of analyses. In Figure 9, the curve shows that the proportion of positive results among all the analyses is not diminished during epidemic years. Neither, the improvement in the diagnostic tools can explain the increase in the reported cases.

**Figure 10:**
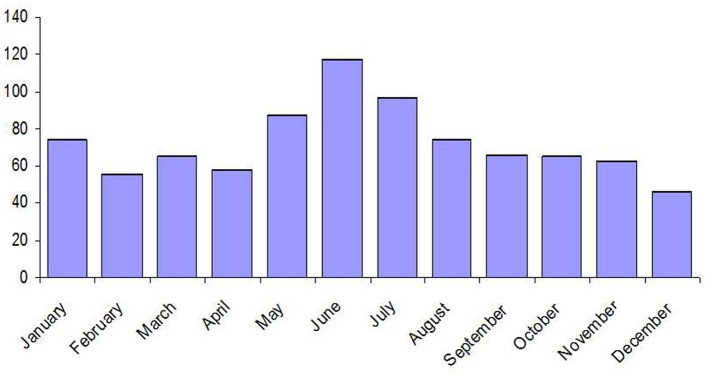
**Distribution per month of the hantavirus cases in France during the period 2001–2009 (data: Institut de Veille Sanitaire, IVS)**.

As PUUV, the most prevalent hantavirus in France, is responsible for a mild form of HFRS, the number of human infections could be under-estimated. Surveys of seroprevalence in endemic areas show that 0.45 of the population has anti-Puumala antibodies. Among people working in the countryside, the overall seroprevalence rate was 1% and even reaching 5% in some areas (Le Guenno, 1997). Most human infections occur during late spring and summer (Sauvage et al., 2002; Vapalahti et al., 2003). For the period 2001–2009, June was the month with the maximum of cases reported, followed by July and May (Figure 10).

Most of the cases are diagnosed in the endemic area (Figure 7) but sporadic cases are reported elsewhere (Figure 7). An infection has been proven using RT-PCR on a Belgian citizen who had been camping near Perpignan, in the Pyrenean mountains, in southern France. After investigations, this patient had no other risk factors than this stay in France (Keyaerts et al., 2004). In the area of Paris, a retrospective study identified 14 cases in 1999 and 2000 (Lautrette et al., 2003).

As previously seen, the risk for human infection is correlated with the prevalence of PUUV in the populations of *M. glareolus* (Salvador et al., 2011). In the Ardennes, the prevalence was 39% in the epidemic year of 2003 while it was 26.5% in 2002 and 29.9% in 2001. In addition, the number of *M. glareolus* trapped was between twice and three times more in 2003 when compared with 2002 and 2001 (Mailles et al., 2005a). As for the contamination within rodent populations, the amount of virus in the environment, linked with the number of recently infected rodents, is critical.

The presence of infected rodents is not the only condition leading to human infections. In an epidemiological survey in the department of the Ardennes, high seroprevalence rates were found in *M. glareolus*, in sites where no human cases were reported (Augot et al., 2008).

Transmission to humans occurs mainly indirectly by inhalation of virus-contaminated aerosols from excreta of infected rodents (McCaughey and Hart, 2000). Human behavior must make such a contact possible. In a Franco-Belgian case-control study, the main risk factors identified were a significant exposure in forests, entry in buildings where rodents are present and living less than 50 m from a forest (Crowcroft et al., 1999).

## Epidemiology of Hantaviruses in Germany

### Brief history on hantavirus infections in germany

In Germany human hantavirus infections are characterized by a mild HFRS and were first published in 1985 (Zeier et al., 1986) followed by sporadic descriptions in the early nineties of the last century (Gärtner et al., 1988; Zeier et al., 1990; Mettang et al., 1991; Pilaski et al., 1991, 1994). An accumulation of hantavirus infections speculating to local hantavirus outbreaks were already postulated for 1983, 1990, and 1993 in different regions in Germany, e.g., in the Swabian Alb, Baden-Württemberg, including also sites for military maneuvers around Ulm (Clement et al., 1996). These human cases were defined by clinical signs of the disease and/or serological evidence for hantavirus antibodies. However, detailed knowledge on the etiological agent such as virus isolates or sequence data on German hantaviruses were not available (Kulzer et al., 1992, 1993; Clement et al., 1994). It still took 10 years until hantaviruses were characterized on the molecular level in Germany, i.e., the PUUV strain Berkel from an HFRS-patient (Pilaski et al., 1994) and strain Erft from a bank vole collected ~70 km away from the area in North-Rhine Westphalia where this patient was infected (Heiske et al., 1999). Finally, in the year 2001 in Germany clinically apparent hantavirus infections became notifiable to official health authorities due to the German Infection Protection Act (Infektionsschutzgesetz, IfSG). By definition, a combination of any of the following symptoms is indicative for HFRS and considered as a case-definition according to the Robert-Koch-Institute (RKI): acute onset of disease with temperatures >38.5°C, back- and/or abdominal pains, headache, proteinuria, and/or hematuria, elevation of serum creatinine levels, thrombocytopenia, and oliguria or polyuria, respectively. The suspected disease is confirmed either serologically or directly through molecular-biological methods. However, the infectious agent can only be detected within the first few days of the disease during the viremic phase using RT-PCR. Thus, a negative RT-PCR result does not necessarily exclude an infection with hantavirus^5^ (accessed January 9, 2012).

### 2001–2011: Changes in the hantavirus epidemiology in germany?

**Figure 11:**
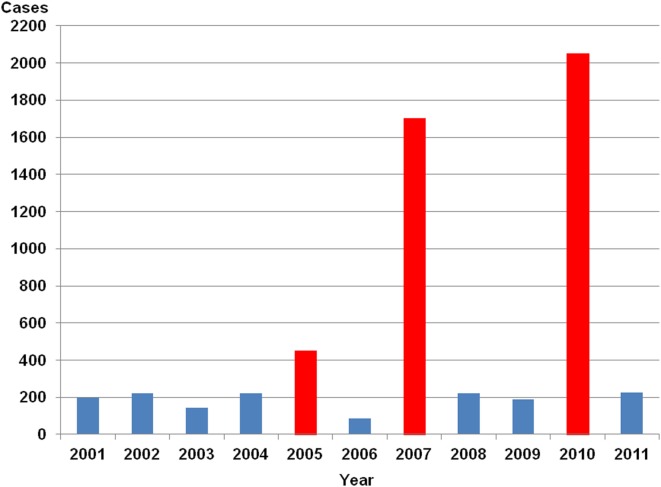
**Yearly number of hantavirus cases in Germany**. Source of data is the SurvStat of the Robert Koch-Institut Data available from http://www3.rki.de/SurvStat, data as of January 25th, 2012, RKI (2012).

**Figure 12:**
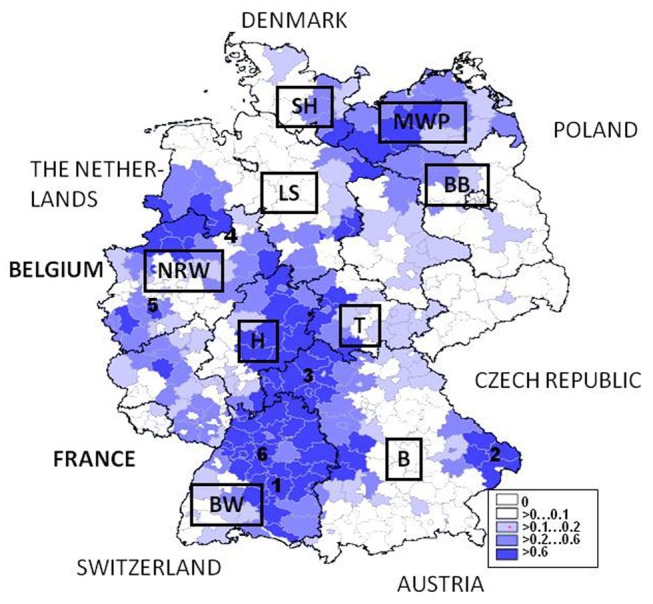
**Germany: map with hantavirus cases/100.000 inhabitants January 1st 2001–January 1st 2012**. Some hot spot regions for PUUV infections are numbered: 1 –Swabian Alb, 2- Lower Bavaria, 3-Main-Spessart, 4-Osnabrück, 5-Cologne, 6-Stuttgart. Source of data is the SurvStat of the Robert Koch-Institut Data available from http://www3.rki.de/SurvStat, data as of January 25th, 2012, RKI (2012). MWP, Mecklenburg-Western Pomerania; BB, Brandenburg; LS, Lower Saxony; SH, Schleswig-Holstein; NRW, North-Rhine Westphalia; H, Hesse; T, Thuringia; BW, Baden-Wuerttemberg; B, Bavaria.

Since the beginning of the mandatory notification, approximately 200 HFRS cases are counted in non-epidemic years with an average incidence of 0.25 per 100,000 inhabitants. Local outbreaks have been reported in 2004 in the federal state of Bavaria (Essbauer et al., 2006; Schilling et al., 2007) and in 2005 (Mailles et al., 2005b; Heyman et al., 2007) affecting regions in Lower Saxony (Ulrich et al., 2008), North-Rhine Westphalia (Essbauer et al., 2007), and Bavaria (Mertens et al., 2009, 2011c). In 2007 a large outbreak occurred with 1,688 registered cases affecting the federal states Baden-Württemberg, Bavaria, Lower Saxony, and North-Rhine Westphalia (Heyman et al., 2007; Koch et al., 2007; Hofmann et al., 2008; RKI, 2012). Further, in the year 2010 a record number of 2,017 cases was reached with outbreaks in the same federal states as in 2007 including also parts of Hesse (Faber et al., 2010; Heyman et al., 2011; Ettinger et al., 2012; RKI, 2012). The summary of annual cases is shown in Figure 11. As described for France there exist some hot spot regions in Germany, i.e., several parts of Baden-Württemberg including the Swabian Alb, in Bavaria the Main-Spessart region and Lower Bavaria, in Lower Saxony the administrative district of Osnabrück (see Figure 12).

Changes in the epidemiology of clinically apparent hantavirus infections in Germany therefore are characterized by a tremendous rise of cases in years 2005, and most notably in 2007 and 2010 but also by an appearance in so far unrecognized regions or in new urban areas. As already discussed in general a changed risk for hantavirus infections for humans may also be inferred from a change in behavior as the working population tends to shift recreational activities to natural settings. In 2005 an unusual outbreak in the cities of Cologne, Aachen, and Osnabrück were recognized (Abu Sin et al., 2007; Essbauer et al., 2007). In Cologne human cases were found close to a wooded city recreation area, an adjacent tennis park, and football stadium. In the forest city park, PUUV was found in high prevalence in the bank vole population and therefore some risk for human infections persists in this area (Essbauer et al., 2007; Ulrich et al., 2008). In 2010 in the urban district of Stuttgart even 166 cases were registered (RKI, 2012).

**Figure 13:**
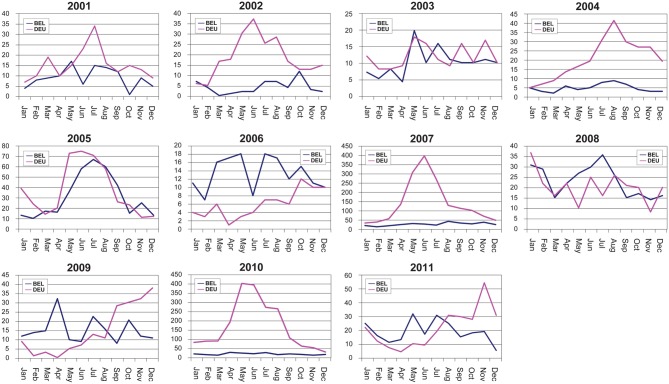
**Monthly oscillations of clinically apparent hantavirus cases in Belgium and Germany, 2001–2011**.

**Figure 14:**
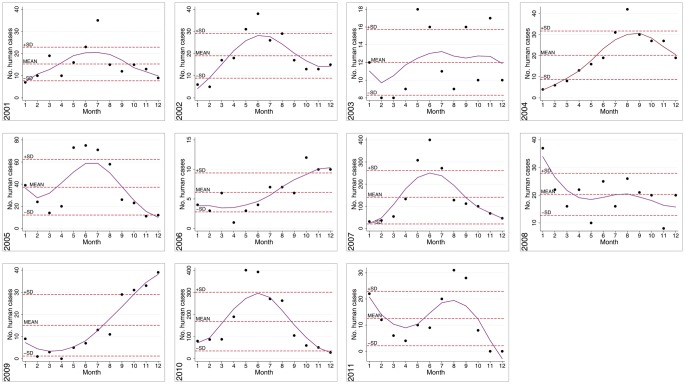
**Seasonal/Monthly oscillations of clinically apparent hantaviruses in Germany, 2001–2011**. Notified human PUUV cases by year were charted to month of notification (1 = January, 2 = February, etc.). Mean monthly cases per year ± SD and yearly smoothed trend lines are shown.

What do we know about seasonal patterns of hantavirus infections in Middle Europe? In general, Germany and other western European countries usually (2001, 2002, 2004, 2005, 2007, 2010) see a summer peak in human PUUV cases. Figure 13 shows the seasonal oscillations of hantavirus infections in Belgium and Germany illustrating the high variability of seasonal peaks for these two neighboring countries. This is in contrast to Fennoscandia (Finland and Scandinavia) where autumn and winter peaks occur (Piechotowski et al., 2008; Evander and Ahlm, 2009). However in Germany in 2006 (very few cases), 2008, and 2009 winter peaks were obtained. Further, in the years 2003 (roughly, very few cases) and 2011 winter and summer peak were observed. In 2010 health authorities were aware of the steep rise of human infections in the winter months and early spring in the year and it was hypothesized that bank voles might move closer to human housings in winters with extreme cold conditions (Faber et al., 2010). In summary, these data indicate a quite complex seasonal pattern of clinically apparent hantavirus infections for Germany that also might be regionally variable (see Figure 14).

### Hantavirus species and rodent hosts

The picture of hantavirus species present in Germany seems to be more complex in comparison to Belgium and France or also Scandinavian countries. As indicated until the beginning of the century only few data on circulating strains were available (Pilaski et al., 1994; Heiske et al., 1999). Several species have been reported so far, yet PUUV found in human patients and bank vole populations in South and West Germany is the predominant virus. Since 2004 intense studies on hantaviruses in their natural reservoir the small mammals have been started in outbreak regions of human infections. Comparable to the French data shown in Table 1, in Germany PUUV-prevalences in affected rodent populations might be quite high reaching 30 up to 60% prevalence in outbreaks (Essbauer et al., 2006; Ulrich et al., 2008; Mertens et al., 2011a). Presently, at least eight geographic and phylogenetic distinct clusters predominate in Germany (Essbauer et al., 2007; Hofmann et al., 2008; Ulrich et al., 2008; Mertens et al., 2011c; Ettinger et al., 2012). Meanwhile, in order to understand the oscillation of human cases intense longitudinal investigations of rodents in several areas have been initiated (Ulrich et al., 2008; Mertens et al., 2011c). Secondly, DOBV is present in Germany (Meisel et al., 1998, 2006; Klempa et al., 2004; Schlegel et al., 2009). Since 2001 approximately 30 human infections with DOBV were diagnosed in Northeast Germany (RKI, 2012). The usually *Apodemus agrarius*-associated strain DOBV-Aa (“Saarema”) was found in *A. flavicollis* in the Federal States of Mecklenburg-Western Pomerania, Lower Saxony, Brandenburg, and also in Schleswig-Holstein (Schlegel et al., 2009; RKI, 2012).

Thirdly, mild human TULV infections were discussed but the definite role of this virus for human infections is quite unclear (Schultze et al., 2002; Klempa et al., 2003). As already mentioned for France multiple vole hosts such as *Microtus agrestis*, *M. arvalis*, and *Arvicola amphibius* were shown to be reservoirs (Schmidt-Chanasit et al., 2010; Schlegel et al., 2012). TULV was found to be present in several geographic regions in Germany, i.e., Baden-Württemberg, Bavaria, Lower Saxony, and Brandenburg (Mertens et al., 2011b). Its evolution seems to be not host-related but several different genetic geographic clusters were shown (Schmidt-Chanasit et al., 2010; Schlegel et al., 2012). Concisely, intense studies on hantaviruses in insectivores have been initiated without knowing the impact of these viruses on human health (Schlegel and Ulrich, personal communication).

### Risk groups and factors for human population

The data as hitherto describe clinically apparent hantavirus infections in humans and some aspects of viruses in rodents. However, the officially reported human cases only present the tip of an iceberg as many infections progress with unspecific symptoms and therefore might not be detected. Seroprevalence studies in the human population and different risk groups were started in the early 1980s in order to understand the “true” impact of hantavirus infections on humans. During these studies serological diagnostics started with classical immune fluorescence assays and were in recent years improved by implementing ELISAs and Western Blots with homologous antigens (Zöller et al., 1993, 1995; Razanskiene et al., 2004; Meisel et al., 2006; Mertens et al., 2009, 2011b). The first detailed seroepidemiological investigation was performed on 13,358 human sera (Zöller et al., 1995), and followed by further studies that showed a general seroprevalence in Germany of approximately 1–2% with higher prevalences (2%) found in South-West Germany (Martens, 2000; Kimmig et al., 2001; Mertens et al., 2009). In endemic regions the hantavirus seroprevalence may locally be quite variable and reach up to 5–10% as it was, e.g., shown in Baden-Württemberg (Zöller et al., 1995; Kimmig et al., 2001) and some communities in Lower Bavaria (Mertens et al., 2009, 2011c; Essbauer, personal communication). Due to their occupational exposure to small mammals and their excreta, several professions are at a higher risk for hantavirus infections, i.e., soldiers (Antoniadis et al., 1985; Clement et al., 1996; Mertens et al., 2009), construction workers (Abu Sin et al., 2007), muskrat hunters (Zöller et al., 1995), workers at horse breeding farms (Zöller et al., 1995), farmers (Rieger et al., 2005), lumberjacks, and woodsman (Zöller et al., 1995; Rieger et al., 2005; Mertens et al., 2011b). The most recent seroepidemiological study in Brandenburg showed that forest workers there have higher TULV and DOBV seroprevalences concluding that both virus types might be predominant and possibly underdiagnosed in this region (Mertens et al., 2011b).

In order to define further risk factors for the human populations as in Belgium and France some further epidemiological studies were performed in Germany. After the 2005 outbreak two case-control studies were initiated. In the area of Osnabruck for 30 eligible hantavirus patients registered between August 2004 and 2005 and a control-group of 43 persons it was shown that staying or living close to a forest, leisure activities in a forest such as camping, jogging, going on a mushroom foray are significant risk factors (Siffczyk et al., 2006). In comparison, a German-wide case-control study was performed between May and August 2005 with 215 appropriate patients and 150 matched controls participating. Living close to forested areas, and noticing mice, but not leisure activities in a forest was found to be a risk for human PUUV infections (Abu Sin et al., 2007). A further study after the 2007 epidemics showed with 191 matched case-control pairs (April to June 2007) in Baden-Württemberg that participants were more likely to have visited a forest and to have seen small rodents or their droppings. Besides, cleaning utility rooms such as sheds, attics, cellars, and garages, or visiting forest shelters in order to make a barbecue or to shelter from the rain were additional risk factors (Winter et al., 2009). In summary, risk factors for Belgium, France, and Germany are defined by contact to nature and rodents but might to some degree depend on the study sites, the annual and local epidemic situation, and the questions asked in the survey.

### Factors driving hantaviruses (or not) in germany

So far the epidemiology and some risk settings for humans acquiring a hantavirus infection have been summarized for Germany. However, investigations of factors, which drive human hantavirus infections’ oscillations such as the prevalences of the virus in rodent populations, the environmental, faunistic, floristic, weather (climatic), and other parameters are still at an early stage. This also includes factors that might have a direct impact on the virus stability in the environment. In general, Germany consists of a broad variety of landscapes formed by former glaciers. In the North these include the Islands of the North- and Baltic Sea, the North German Plain. In Central Germany uplands and numerous river valleys, e.g., Rhine are present. Southern Germany is characterized by several linear hills and high mountain ranges such as Swabian and Franconian Alb, Bohemian Forest, and the Alps. There are three main biogeographic regions, the Atlantic, the continental-middle European, and the alpine (Glaser et al., 2007). Hence, there exist a broad variety of habitats and biotopes for rodents and makes the situation for these animals and therefore associated diseases quite complex. In 2004 a network on rodent-associated pathogens with an intense focus on hantaviruses was established in order to bring scientist with different expertise together to investigate aforesaid issues (Ulrich et al., 2008). Several studies are presently performed, e.g., financed by the Robert-Koch-Institute, the Federal German Environmental Agency, the Bavarian State Ministry of Environment and Public Health^6^, or in parts by the EEC (EDENnext^7^). Details of this network and actual projects have been actually summarized recently by (Rosenfeld, personal communication).

In the following subchapter actual data on factors driving hantaviruses in Germany available from the literature are reviewed. In part, so far unpublished results from a 3-year-study in the German hantavirus hot spot region Bavarian Forest National Park in Lower Bavaria are presented (see marked in Figure 12). Here from 2008 to 2010 small mammals and their associated pathogens (e.g., PUUV and *Rickettsiae*; Schex et al., 2011; Silaghi et al., 2012) were analyzed along an altitude gradient together with environmental, i.e., vegetation and climatic data generated in the BIOKLIM project (Biodiversity and Climate Change Project; Bässler et al., 2009). In brief, data from 661 small mammals including 275 bank voles trapped at 23 sites were used in order to analyze if rodent-associated factors, i.e., biometric, diversity, environmental, and climatic or weather conditions influence the prevalence of PUUV in lower Bavaria in order to reflect the risk of infections for the human population (Thoma, personal communication).

### Biodiversity

As mentioned earlier in this review biodiversity may influence host-pathogen interactions and thus the prevalence of infectious agents leading to the emergence/re-emergence of diseases. However, the role of biodiversity on pathogen pattern changes is actually controversially and extensively discussed (Maillard and Gonzalez, 2006; Aguirre and Tabor, 2008; Maillard and Sparagano, 2008). While the hypothesis that high species diversity decreases pathogen prevalence through mechanisms such as decreased host density, reduced encounters between hosts, alterations in host behavior or reduced host survival (“Dilution Effect”) commonly exists another theory is postulated (“Amplification” or “Rescue Effect”) where increased pathogen prevalence is associated with greater species diversity through increased encounters between hosts, or through the presence of secondary hosts (Keesing et al., 2006). The “Dilution Effect” has been demonstrated for pathogens other than hantaviruses, i.e., for Leptospirae it was shown that a reduced mammal species biodiversity increased its incidence (Derne et al., 2011). Similar observations have been made for other rodent-borne viruses (Mills, 2006) including also several New World hantaviruses. In example, for Sin Nombre, Choclo, and Laguna Negra virus a negative relationship between species diversity and hantavirus prevalence has been emphasized (Yahnke et al., 2001; Ruedas et al., 2004; Dizney and Ruedas, 2009). In Belgium the PUUV prevalence in *M. glareolus* was negatively affected by the relative proportion of the non-host *A. sylvaticus* (Tersago et al., 2008). Further, for Sin Nombre Virus there is also experimental evidence for the “Dilution Effect,” which has been demonstrated through manipulation of small mammal biodiversity in wild reservoir populations in Panama (Suzán et al., 2009). In contrast to the above studies, for PUUV in Lower Bavaria, where *M. glareolus*, *A. flavicollis*, *A. sylvaticus*, *M. agrestis*, and *Sorex* spp. are predominant, the association of reduced rodent diversity and higher prevalence of PUUV could not be confirmed (Thoma, personal communication). Here, increased species diversity was correlated with higher PUUV estimates in the host rodent, which is in line with the “Amplification Effect.” With regard to this phenomenon several hypotheses may be formulated: (i) species other than the primary host could have the ability of functioning as a further reservoir for PUUV and thus lead to an increased transmission of the virus and overall prevalence; (ii) the presence of a species with a low reservoir capability could lead to higher contact rates between the primary hosts and subsequent higher transmission of the virus (Clay et al., 2009). Nevertheless, whereas biodiversity may play a key role in pathogen transmission further factors which drive regional differences must exist.

### Landscape and habitat factors

Landscape attributes might have a beneficiary effect on rodent host population dynamics. There exists excellent data on the influence of topography and soil properties on dynamics of common voles from almost 40 years collected in eastern Germany. The analyses showed for example that mean elevation, area-related percentage of Chernozem soils, and soil air capacity are variables that have an influence on the common vole outbreak risks at different sites (Blank et al., 2011). The link to forest environments has been discussed in earlier chapters. More precisely, deadwood and denser ground vegetation, which are found both in forest reserves and managed forests to varying degrees, may serve as a microhabitat and deadwood is used by many species as a food resource, breeding habitat, and for shelter. Further, species richness correlates high with deadwood volume (Siitonen, 2001; Maguire, 2002). Deadwood thus represents an important feature and may therefore relate to higher bank vole population densities, which may increase the overall hantavirus prevalence in the rodent host, and provoke hantavirus epidemics (Rooney and Hayden, 2002). Analogous to deadwood, dense ground vegetation may also serve as an ideal habitat for bank voles and has been associated with PUUV foci in other countries (Escutenaire et al., 2002; Tersago et al., 2008). In Lower Bavaria, increasing percent coverage of deadwood and dense ground vegetation significantly increases the probability of PUUV prevalence in *M. glareolus* (Thoma, personal communication). In conclusion, despite the important aspects of deadwood for a healthy ecosystem and the tendency to launch such traditional and untouched forests enhanced deadwood may be one factor to increase a risk for PUUV infections.

### Mast

Mast already was defined in detail and critically discussed above in the overview on PUUV in Belgium. As described in detail for Belgium criteria for mast may be very versatile. The comparison of the seasonality of human hantavirus infections in Belgium and Germany (Figure 13) also demonstrates that oscillations in these two countries are quite variable and therefore a general conclusion on mast, rodent populations, and hantavirus infections should be avoided. In Germany an annual report on the conditions of German forests (“Waldzustandsbericht”) is published from the Federal Governmental Ministry of Consumer Protection, Food, and Agriculture (e.g., for 2007^8^). The report includes an overview of the status and also damages on different trees but does not include concrete data on mast. Additionally, in Germany certain officers responsible for forest and agriculture from each federal state meet once a year in order to exchange data on mast, rodent damages, and actual prognostic trappings. Despite the importance of this work in recent years, manpower, temporal, and financial possibilities have been reduced. In general, differences on results of mast of different trees, rodent populations, and also at the investigated sites are tremendous and therefore cannot be compared with the conditions in Scandinavia. The results of these meetings are embedded in the network rodent-borne diseases in order to combine the different expertise and to communicate the data also to health offices (Ulrich et al., 2008).

As already mentioned in the chapters above, in principal food availability (as, e.g., beechnut mast) has an influence on population dynamics of bank voles. In contrast to other rodent species (e.g., *M. agrestis* and *M. arvalis*) bank voles may be more dependent on tree seeds such as beechnut. Data from Lower Saxony from 16 years on beech fructification and the abundance of bank voles showed that the fruits have a positive effect on the population in the next year. For negative trends the context between fruit and vole was true in 75% of the cases (Kühn et al., 2011). As beech forest might be beneficial habitats for this species and populations may reach high densities some investigations were performed in Baden-Württemberg. There highest beech forest cover was found in the Swabian Alb (17.7%) and lowest cover in the eastern and middle regions and major cities of Baden-Wuerttemberg (1.6%). Data on annual mast production of beechnuts was obtained between 2000 and 2006. Cumulative incidence (2001–2007) of PUUV infections in Baden-Wuerttemberg is reported highest in the Swabian Alp region. Highest maximum incidence rates for human PUUV cases ranged from 2.28 per 100,000 population in 2007 to 1.16 per 100,000 population in 2006, which is considered a year with beech crop failure (Schwarz et al., 2009). For the year 2010 a growth of the bank vole rodent population could be expected as at two study sites in Swabian Alb and around Münster there was an enhanced beechnut mast in 2009 (Jacob and Ulrich, personal communication). However, the 2010 PUUV outbreak in Germany again showed significant local differences. In conclusion, there exists no enough valid, longitudinal collected and critically summarized data on mast, rodent populations, and associated diseases in Germany as it was also discussed for Belgium and France.

### Climate

Germany is characterized by a warm temperate humid mid-latitude climate with predominating westerly winds carrying humid air masses from the Atlantic Ocean. This influence weakens from the northwest to the southeast. In the northwest and the north the climate is extremely oceanic and rain falls all the year round. Winters there are relatively mild and summers comparatively cool. In the east the climate shows clear continental features; winters can be very cold for long periods, and summers can become very warm. Dry periods are often recorded. Special effects occur in the eastern parts of South Germany especially bringing summery weather conditions during the winter half year, occasionally^9^ (accessed January 4, 2012). Only few analyses on the influence of weather data on rodent populations and rodent-transmitted agents are available in Germany.

Mild winters and springs may positively influence rodent survival rates and early food supply and thus affect rodent population dynamics. The density of the rodent population may well drive the distribution and prevalence of disease/associated agents in the rodent host. For *M. arvalis* populations in the Eastern Federal States of Germany the impact of weather parameters in winter and early spring was revealed to be of some importance for outbreaks in the autumn. Data was collected over almost four decades but also indicated that there may exist regional differences of the impact of weather and that winter and early spring records only may manipulate extremely high and low outbreak risks (Imholt et al., 2011). These results again indicate the complexity of the system and that not only one group of parameters might stimulate rodent populations and therefore associated pathogens. Besides for PUUV carried by bank voles some efforts have been started in order to understand the impact of weather conditions or climate on the oscillations in Germany. Data from 2001 to 2007 from Baden-Wuerttemberg showed that in 2001, 2003, 2004, and 2007 winter temperatures were above long-term averages and in 2001, 2002, 2003, 2004, and 2007 spring temperatures exceeded long-term averages. The maximum winter temperature deviation was recorded in 2007 when reported human hantavirus cases in Germany rose to over 1,500 cases. In marked contrast, in 2006 winter temperatures were lowest as were cases of NE reported in Baden-Wuerttemberg (*n* = 17). Interestingly, as compared to other years between 2001 and 2007, in 2005 there also seemed to be a beech crop failure (Piechotowski et al., 2008; Schwarz et al., 2009). Human hantavirus prevalences may further be driven by the fact that human contact with rodent host abundance is increased when humans shift their recreational activities to bank voles’ environments during mild weather conditions, preferably in early summer (Schwarz et al., 2009). Fluctuations in common vole population densities and thus prevalence of hantavirus in the rodent host may also be correlated with December snowfall, January sunshine duration, and snow fall in April (Ulrich et al., 2008). Interestingly, as opposed to findings in Baden-Wuerttemberg in Lower Bavaria no significant link between winter and spring temperatures and elevated hantavirus prevalences could be corroborated, which again highlights the complex nature of hantavirus disease transmission (Thoma, personal communication).

As described earlier the viability of a hantavirus outside the host may be dependent on climatic factors such as temperature and moisture. Yet, additional factors such as UV radiation may also have an impact on virus tenacity. In experimental studies for appropriate inactivation of Hantaan virus the virus was effectively inactivated through UV irradiation in cell cultures and supernatants (Kraus et al., 2005). Photochemical inactivation of alpha- and poxviruses was demonstrated in another study. Effective inactivation of the viruses was dependent on the type of photochemical used (e.g., 5-iodonaphthyl 1 azide or amotosalen) in combination with UVA (Sagripanti et al., 2011). In the environment, it could be postulated therefore that high values of solar radiation may have an influence on the stability or occurrence of PUUV. In Germany, in the Bavarian Forest National Park in Lower Bavaria, it was shown that after adjusting for confounding factors such as elevation high values of mean annual solar radiation were associated with a decreasing PUUV prevalence in bank voles (Thoma, personal communication). This finding has yet to be shown in other hantavirus hot spots in Germany.

### Rodent specific factors

#### Age

There is good evidence that in the wild PUUV-prevalences in bank voles are age-dependent (Bernshtein et al., 1999; Escutenaire et al., 2002). The age of bank voles is commonly estimated using bodyweight as a proxy as the basis for weight class categories, e.g., juvenile, subadult, and adult (Escutenaire et al., 2002; Kallio et al., 2007). Another method applied for age determination of voles is by assessing the development of the molars (Bernshtein et al., 1999; Olsson et al., 2002). As described for France, age-dependency of virus prevalence in the rodents may be indicative for a horizontal transmission of PUUV in the rodent host (Tersago et al., 2011b). An explanation for this might be that adult rodents will have been exposed to viruses longer than young ones and on more occasions. Another theory discusses that older male rodents may suffer from immunosuppression due to high levels of stress hormones, which are associated with breeding, making them more vulnerable to infections (Deter et al., 2008a). That sexually mature rodents may be at higher risk for infection due to fighting rituals, mating, and generally close social behavior was already mentioned (Escutenaire et al., 2002). Moreover, juvenile rodents will have benefited from maternal antibodies during the first 3 months of their live and therefore less often been infected during suckling (Kallio et al., 2006b, 2010). Findings in Lower Bavaria affirm these observations. In the large part older animals are infected with PUUV. The odds of the tested bank voles being PUUV seropositive constantly increased with each unit increase of the rodents’ bodyweights (Thoma, personal communication). By implication, given the horizontal transmission and as indicated by the respective chapters in this paper climatic and environmental factors may have a benefit on the survival and aging of rodent populations and on population density. Together, increasing rodent population densities may lead to increased rodent contacts, which may foster the transmissibility of the virus, resulting in higher prevalences, and eventually driving and influencing hantavirus epidemics.

Male bank voles are believed to be of greater risk of acquiring and spreading PUUV and thus play an important role in the dynamics of PUUV epidemics (Bernshtein et al., 1999; Olsson et al., 2002; Deter et al., 2008a). *R. norvegicus* and *Calomys musculinus* show a similar pattern regarding transmission of SEOV and Junin virus, respectively (Mills and Childs, 1998). Especially in the reproductive season male animals are very mobile and have close contacts with other members of their species. The effects of aggression and wounding due to sexual activity of male animals during mating season on the dissemination of disease have been discussed, which may be considered as a driving force for hantavirus epidemics (Bernshtein et al., 1999; Olsson et al., 2002; Deter et al., 2008a; Tersago et al., 2011b). In contrast to this observation, in rodents investigated in the Bavarian Forest National Park in Lower Bavaria the PUUV prevalence levels in males were not significantly higher than in females (Bernshtein et al., 1999; Olsson et al., 2002; Deter et al., 2008a). These findings suggest that there is at least in that region no gender specific affinity to the virus. However, local genetic differences in the bank vole population in this region may produce subpopulations, which render differently and gender-independent with regard to susceptibility to the virus. Various patterns of PUUV infection and resistance (tolerance) depending on different genetic variants of the TNF-α locus were for example discussed for bank voles in France and Germany (Guivier et al., 2010). With regard to other hot spot regions in Germany no reliable data on gender exist and has yet to be shown in further longitudinal studies. Again, these findings highlight the complex nature of driving factors for hantavirus transmission and host ecology.

## Conclusion

Our findings indicate that the occurrence of clinically apparent human hantavirus infections in Belgium, France, and Germany are highly variable in time and space. Comparing the epidemiological features in the three forenamed countries also indicated that hantavirus outbreaks in our three countries are also not always triggered by the same factors.

We defined six major groups that could act as triggers or regulators of hantavirus outbreaks in Western Europe; behavioral factors of reservoir and host, landscape and changes in land-use, climate, food availability, biodiversity, and physiological features of the reservoir population. Care should however be taken not to generalize the relationships as some have already been proven wrong in the past (Brown and Ernest, 2002).

The ever-present pitfall however, is relating the occurrence of infectious diseases in general and hantavirus infections in particular to one particular trigger, e.g., food availability. Climate change, for instance, is a likely facilitator but climate change is an ancient and ongoing process (Ruddiman, 2003) that we experience through a narrow window in time. The environment seems a vital intermediary factor when it concerns pathogen transmission between carrier and host; which points to the possibility that the transmission rate is not only determined by carrier abundance or pathogen prevalence, but also by rodent ecology, virus survival, local climatic conditions, and human behavior (Lambin et al., 2010). We have for most of the regulating factors – unfortunately – hardly an idea where we come from and where we are going to in this matter. Relationships between abiotic and biotic parameters, rodents, humans, and hantavirus infections are first and foremost highly complex and highly intertwined but – should we be able to evaluate them accurately – can be valuable for predicting hantavirus epidemics.

## Conflict of Interest Statement

The authors declare that the research was conducted in the absence of any commercial or financial relationships that could be construed as a potential conflict of interest.

## Footnotes

^1^http://www.iucnredlist.org/

^2^http://www.enivd.de/FS/fs_encdiseases.htm

^3^http://www.meteo.be

^4^www.wilde-planten.nl

^5^www.rki.de

^6^http://www.lgl.bayern.de/das_lgl/forschung/forschung_interdisziplinaer/fp_vicci_project5_en.htm

^7^www.eden-fp6project.net

^8^http://www.bmelv.de/cae/servlet/contentblob/383616/publicationFile/22185/ErgebnisseWaldzustandserhebung2007.pdf

^9^www.dwd.de
